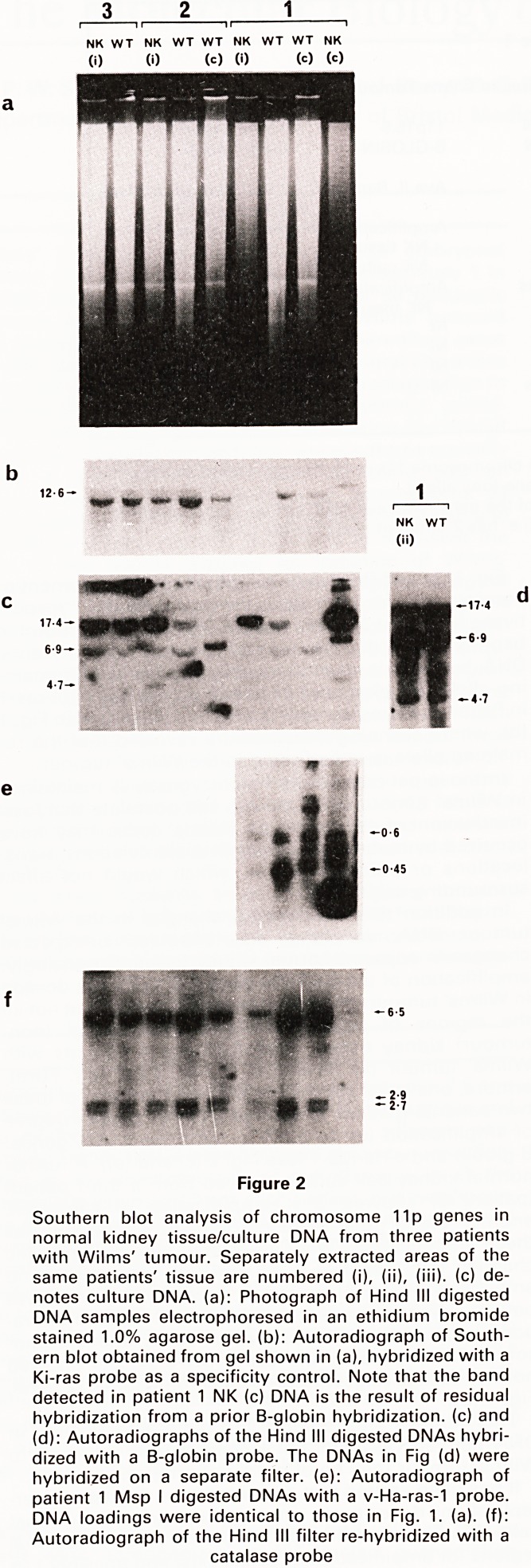# Molecular Biology of Wilm's Tumour

**Published:** 1988

**Authors:** A. P. W. Shaw, V. Poirier, S. Finerty, P. J. Berry, M. G. Mott, N. J. Maitland

**Affiliations:** Department of Pathology, University of Bristol Medical School; Department of Pathology, University of Bristol Medical School; Department of Pathology, University of Bristol Medical School; Department of Pathology, University of Bristol Medical School; Department of Pathology, University of Bristol Medical School; Department of Pathology, University of Bristol Medical School


### The Molecular Biology of Wilms' Tumour

**A. P. W. Shaw, V. Poirier, S. Finerty, P. J. Berry, M. G. Mott and N. J. Maitland**
Department of Pathology, University of Bristol Medical School

### INTRODUCTION

Wilms' Tumour (Nephroblastoma) is an embryonal tumour of the kidney, which affects approximately 1 in 10,000 children and accounts for 6% of all paediatric cancers. Although the vast majority of Wilms' tumours are sporadic, in the small percentage of hereditary cases (4–8%) observed, the tumour is often bilateral and arises at an early age. Furthermore, a genetic predisposition to develop the tumour is associated with aniridia, genito-urinary abnormalities and mental retardation (the WAGR syndrome) (1). Children with this rare syndrome typically carry a germline deletion involving band p13 on one of the two (parentally-derived) chromosome 11 homologues (2). The inherited 11p deletion in WAGR and hereditary Wilms' patients is thought to represent the first of two events required for initiation of Wilms' tumour, as postulated by Knudson from epidemiological studies (3). In addition, the specific loss of chromosome 11p alleles has been shown in sporadic Wilms' tumours (4–7). This loss of normal cellular 11p sequences in Wilms' tumourigenesis, correlates with the principle of development of somatic homozygosity of a recessive defect within 11p13. It has consequently been postulated that the intact wild-type Wilms' tumour locus at 11p13 may encode a tumour suppressor and/or differentiation function (8). Interestingly, the same pathogenetic mechanism involving the 11p13 locus is implicated in other closely related childhood tumours: hepatoblastoma, rhabdomyosarcoma and adrenal carcinoma (8).

This study concerns an analysis of Wilms' tumour, adjacent normal kidney and blood cell DNA from 6 sporadic Wilms' tumour patients, for any chromosome 11 gene changes which might be related to tumour development.

### METHODS

*Subjects*: The six patients studied were between 2.5 and 6 years old with unilateral sporadic Wilms' tumour and no evidence of aniridia. Tumour and adjacent non-tumour kidney tissue were obtained at tumour resection, prior to any chemotherapy or radiotherapy. In all the specimens examined from each patient, this neighbouring non-tumour tissue was histologically normal. Small pieces of fresh tumour and adjacent kidney were explanted on to mitomycin C treated 3T3 feeder cell monolayers and any cell cultures obtained were maintained for up to 9 passages with feeders and Dulbecco's modification of Eagle's medium supplemented with 15% fetal bovine serum, hydrocortisone (1 ug/ml), insulin (0.2 units/ml), and epidermal growth factor (10 ug/ml) (9). Where possible blood samples were also obtained and the leukocytes transformed with Epstein-Barr virus (EBV) to establish lymphoblastoid B cell lines as a permanent source of constitutional DNA (10).

*DNA Extraction*: Tissue fragments and pelleted culture cells were homogenized in 4M (molar) guanadinium isothiocyanate, layered onto a two-step caesium trifluoroacetate density gradient (density=1.75 and 1.5 g/l) and the nucleic acid prepared by ultracentrifugation at 40,000 rpm for 16 hours, with subsequent repeated phenol/chloroform extraction (modified from Chirgwin et al (10).

*Southern blot analysis*: High molecular weight DNAs (10 ug) were cleaved with the appropriate restriction enzymes and transferred to Hybond-N membranes (according to Amersham protocol). These Southern blots were hybridized with four chromosome 11p $^{32}$p-oligolabelled (12) DNA probes (catalase (11p13), calcitonin, B-globin and c-Ha-ras-1 (all 11p15)) of specific activity>$10^8$ cpm/ug, under conditions of moderate stringency: 33% formamide, 2×SSC (1×SSC=0.15 M sodium chloride, 0.015 M sodium citrate, pH 7.0) for 18 hours at 45°C.

*Post-hybridization washing and autoradiography*: Hybridized blots were washed in 2×SSC+0.5% sodium dodecyl sulphate (SDS) for 2 hours at 65°C, and then exposed to X-ray film between intensifying screens at −70°C for 1–3 days. Autoradiographs were analysed by scanning densitometry (Bio-Rad model 620, video densitometer) to allow quantitative comparison between Wilms' tumour and normal kidney/B cell DNA.

### RESULTS AND DISCUSSION

If DNA is digested with appropriate restriction enzymes (which cleave the DNA at specific short sequences) and Southern blot analysis performed with chromosome 11 DNA probes, it is possible to distinguish the maternal/paternal chromosome homologues (alleles) of a gene by means of restriction fragment length polymorphisms (RFLPs). There are natural variations in base sequence (in and around genes) between individuals, due to point mutations or to the presence/deletion of short repetitive sequences. These natural variations in base sequence will generate changes in the length of the restriction fragment on which the gene of interest is located. Changes in fragment length can then be detected with the particular gene probe, i.e. as restriction fragment length polymorphisms (13).

Some of the most important mechanisms by which it might be possible for the normal Wilms' tumour locus to become defective or absent on both chromosome 11s in the tumour (namely mitotic nondisjunctional loss of a chromosome with or without reduplication, and mitotic recombination) also bring about similar changes in other genes on the same chromosome. Thus whilst the Wilms' tumour locus itself is not defined, we can predict loss at this locus by exploiting RFLPs to analyse for loss of alleles of other genes on the same arm of chromosome 11. In common with other workers (4–7), we find that in patients where DNA from normal kidney tissue/blood is heterozygous (i.e. 2 different RFLPs corresponding to 2 different alleles are detectable), the Wilms' tumour DNA is often hemi- or homozygous (i.e. the tumour cells

**Table 1**

**Summary of Analysis of Chromosome 11 genes in Wilms Tumour DNA from 6 patients**

| | | | | |
|---|---|---|---|---|
| *Location:* | 11p13 | 11p15.1–15.4 | 11p15.5 | 11p15.5-pter |
| *Probe:* | CATALASE | CALCITONIN | B-GLOBIN | C-HA-RAS-1 |
| *Enzymes to detect RFLPs:* | Ava II, Kpn I and Hae III | Taq I | Ava II, Bam HI | Taq I, Bam HI, & MspI/ HpaII |
| *Patient 1* | NI | NI | Amplification in NK tissue 6x, NK culture 29x | Heterozygous |
| *2* | Heterozygous | Heterozygous | Amplification in NK tissue 3.7x | NI |
| *3* | Hemizygous | Hemizygous | NI | Hemizygous |
| *4* | Heterozygous | NI | NI | NI |
| *5* | Homozygous | NI | NI | Homozygous |
| *6* | Homozygous | Homozygous | NI | NI |

NK= Normal kidney
NI= non-informative; NK/B cell DNA is homozygous (both chromosome 11s carry the same allele of the gene)
Hemizygous= Wilms' tumour has only one copy of the gene (one allele)
Homozygous= Wilms' tumour has 2 copies of one allele of the gene, whereas NK/B cell was heterozygous
Heterozygous= Wilms' tumour maintains heterozygosity (i.e. has 2 different alleles of the gene)

contain only one copy of a gene (one allele): hemizygosity, or that the tumour cells have lost one allele and reduplicated the remaining allele to give 2 copies of one allele: homozygosity). In fact, in 3 of the 6 patients studied we have been able to detect this loss of heterozygosity, with at least one chromosome 11p gene probe (as summarized in Table 1 and partially illustrated in Fig. 1).

a b

**Figure 1**

Southern blot analysis for loss of 11p heterozygosity in Wilms' tumour DNA from five patients. NK: denotes normal kidney tissue DNA; WT: denotes Wilms' tumour tissue DNA; B: denotes EBV-transformed lymphoblastoid B cell DNA. The size of each hybridizing fragment is given in kilobases (kb) alongside each figure. (a) Autoradiograph of TaqI digested DNA hybridized with a calcitonin probe (two RFLPs are detected here). Patient 3 shows development of hemizygosity and patient 5 of homozygosity in WT DNA

Fig. 1 (a) and (b) clearly illustrates the development of hemizygosity for both calcitonin and c-Ha-ras-1 respectively, in patient 3 Wilms tumour DNA. One polymorphic band is lost in the tumour compared to normal kidney DNA, but there is no simultaneous duplication of remaining allele. Development of homozygosity for c-Ha-ras-1 in Patient 3 Wilms' tumour is similarly illustrated in Fig. 1 (b), where scanning densitometry revealed that the remaining allele is reduplicated in the Wilms' tumour.

In those patients where heterozygosity is maintained in Wilms' tumour DNA, we can still postulate that loss/ inactivation of the wild-type Wilms' locus may have occurred by means of very small scale deletions, translocations or point mutations, which would not affect surrounding sequences.

In addition to characterizing changes in the Wilms' tumour DNA, we have also observed unexpected changes in adjacent normal kidney tissue. Surprisingly, amplification of the B-globin gene (up to 6×the dosage in Wilms' tumour DNA) was observed in some but not all the regions of adjacent histologically normal (non-tumour) kidney tissue, from 2 of the 6 patients with Wilms' tumour (as illustrated in Fig. 2 (c), (d)). Furthermore, one renal cell culture derived from one of these two patients (patient 1) showed an even greater degree of amplification (up to 30×) for both the 11p15 genes: B-globin and c-Ha-ras-1 (see Fig. 2 (c) and (e)). A further normal kidney cell culture derived from a third patient (patient 6) showed 3 × amplification of calcitonin, another 11p15 gene (data not shown). In the cultures these amplifications were associated with polymorphic changes (e.g. see Fig. 2 (c) and (e)). Importantly, no amplification was observed in B cell DNA from these patients. Neither were they widespread random alterations, because DNA markers examined on other chromosomes by re-probing the same DNA digests showed normal dosage (e.g. with Ki-ras a chromosome 12 gene, Fig. 1 (b)).

This variable and regional 11p gene amplification in DNA derived from normal kidney tissues adjacent to Wilms' tumour may be of great importance.

It has been argued that a spontaneous degree of over-replication does occur in non-tumourigenic normal cells (14). There is evidence that preferred chromosomal regions for amplification of genes exist and possibly 11p

**Figure 2**

Southern blot analysis of chromosome 11p genes in normal kidney tissue/culture DNA from three patients with Wilms' tumour. Separately extracted areas of the same patients' tissue are numbered (i), (ii), (iii). (c) denotes culture DNA. (a): Photograph of Hind III digested DNA samples electrophoresed in an ethidium bromide stained 1.0% agarose gel. (b): Autoradiograph of Southern blot obtained from gel shown in (a), hybridized with a Ki-ras probe as a specificity control. Note that the band detected in patient 1 NK (c) DNA is the result of residual hybridization from a prior B-globin hybridization. (c) and (d): Autoradiographs of the Hind III digested DNAs hybridized with a B-globin probe. The DNAs in Fig (d) were hybridized on a separate filter. (e): Autoradiograph of patient 1 Msp I digested DNAs with a v-Ha-ras-1 probe. DNA loadings were identical to those in Fig. 1. (a). (f): Autoradiograph of the Hind III filter re-hybridized with a catalase probe includes such sites. Certainly 11p15 seems a likely 'fragile site' at which generation of specific chromosome rearrangements may be correlated with cancer (15). In addition, trisomy of 11p15 has been found in somatic cells in Beckwith-Wiedemann Syndrome, where there is a strong predisposition to develop Wilms' and related embryonal tumours (16).

One possible explanation for the specific 11p15 gene amplifications in normal kidney could be that they arise as a consequence of tumour development, perhaps because of factors produced by the adjacent tumour. It would be particularly interesting if the amplification extended to include the intact Wilms' tumour locus, as the adjacent normal kidney might then be viewed as amplifying its own locus (and presumably producing the encoded suppressor factor) in response to the Wilms' tumour development. We have seen no amplification of either catalase or calcitonin when the same filter shown in Fig. 2 (c) was re-hybridized with these probes (e.g. with catalase in Fig. 2 (f)). Nevertheless, amplification of other 11p sequences of importance may be of significance, as yet unknown.

An alternative explanation is that the amplification may be a preceding abnormal genetic event in the kidney which then predisposes to tumour development as a secondary event. Clonal expansion of a single cell containing amplified chromosome 11 material would result in a small discrete region of normal kidney containing the amplification. Certainly, clonal selection *in vitro* for cells containing the amplification is one likely explanation for the increased degree of amplification observed in the culture derived from patient 1 normal kidney.

We would suggest that whilst loss of both normal copies of the Wilms' tumour locus is central to tumourigenesis, other genetic events, such as amplifications, may have an important role in the abnormal developmental pathway that leads to Wilms' tumour.

**ACKNOWLEDGEMENTS**

This work was funded by C.L.I.C.. We would like to thank Mrs S. Tyler for her tissue culture aid; Dr N. D. Hastie for supplying the catalase and calcitonin probes; Professor R. Williamson for the B-globin probe and Dr N. Teich for the Ha-ras and Ki-ras probes. We also thank Mr C. C. Jeal for expert photographic assistance, and Ms J. McRill and Mrs J. Gilbert for typing the manuscript.

**REFERENCES**

1. MILLER, R. W., FRAUMENI, J. F. Jr. & MANNING, M. D. (1964) *New Engl. J. Med.* **270**, 922–927.

2. RICCARDI, V. M., SUJANSKY, E., SMITH, A. C. & FRANCKE, U. (1978) *Pediatrics* **61**, 604–610.

3. KNUDSON, A. G. Jnr & STRONG, L. C. (1972) *J. Natn. Cancer Inst.* **48**, 313–324.

4. KOUFOS, A. et al. (1984) *Nature* **309**, 170–172.

5. ORKIN, S. H., GOLDMAN, D. S. & SALLAN, S. E. (1984) *Nature* **309**, 172–174.

6. REEVE, A. E. et al. (1984) *Nature* **309**, 174–176.

7. FEARON, E. R., VOGELSTEIN, B. & FEINBERG, A. P. (1984) *Nature* **309**, 176–178.

8. KOUFOS, A. et al. (1985) *Nature* **316**, 330–334.

9. RHEINWALD, J. G. & GREEN, H. (1979) *Cell* **6**, 331–334.

10. POPE, J. H., HORNE, M. K. & SCOTT, W. (1968) *Int. J. Cancer* **3**, 857–866.

11. CHIRGWIN, J. M., PRZYBYLA, A. E., MACDONALD, R. J. & RUTTER, W. J. (1979) *Biochemistry* **18**, 5294–5299.
12. FEINBERG, A. P. & VOLGESTEIN, B. (1983) *Anal. Biochem.* **132**, 6–13.
13. BOTSTEIN, D., WHITE R. L., SKOLNICK, M. & DAVID, R. W. (1980) *Am. J. Hum. Genet.* **33**, 314–331.
14. SCHIMKE, R. T., SHERWOOD, S. M., HILL, A. B. & JOHNSON, R. N. *Proc. Natn. Acad. Sci. U.S.A.* **83**, 2157–2161.
15. YUNIS, J. J. & SORENG, A. L. (1984) *Science* **226**, 1199–1202.
16. TURLEAU, C. & DE GROUCHY, J. (1985) *Annals de Genetique* **28**, 93–96.

## Figures and Tables

**Figure 1 f1:**
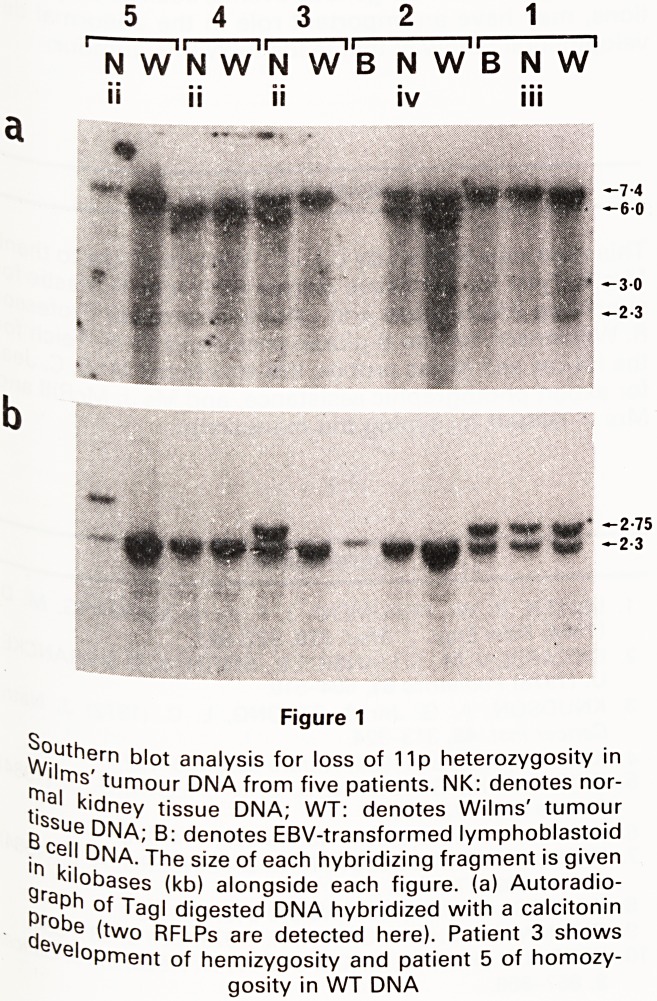


**Figure 2 f2:**